# A new Late Cretaceous metatherian from the Williams Fork Formation, Colorado

**DOI:** 10.1371/journal.pone.0310948

**Published:** 2024-10-23

**Authors:** Jaelyn Eberle, Joshua Cohen, John Foster, ReBecca Hunt-Foster, Andrew Heckert

**Affiliations:** 1 University of Colorado Museum of Natural History and Department of Geological Sciences, University of Colorado, Boulder, Colorado, United States of America; 2 Biology Department, California State University Northridge, Northridge, California, United States of America; 3 Utah Field House of Natural History State Park Museum, Vernal, Utah, United States of America; 4 Dinosaur National Monument, Jensen, Utah, United States of America; 5 Department of Geological & Environmental Sciences, Appalachian State University, Boone, North Carolina, United States of America; New Mexico Museum of Natural History & Science, UNITED STATES OF AMERICA

## Abstract

*Heleocola piceanus*, a new, relatively large metatherian from Upper Cretaceous (‘Edmontonian’) strata of the Williams Fork Formation in northwestern Colorado is described, based on a recently discovered jaw fragment (MWC 9744), in addition to three isolated teeth initially referred by other studies to *Aquiladelphis incus* and *Glasbius piceanus*. Although sharing several morphologic characters with the Lancian genus *Glasbius*, *H*. *piceanus* lower molars are considerably larger than those of *Glasbiu*s and differ from the latter in lacking a buccal cingulid, possessing carnassiform notches on the cristid obliqua and entocristid, and bearing an entoconulid on m3. To examine the relationship of *Heleocola piceanus* to other metatherians, *H*. *piceanus* was scored into a previously existing taxon-character matrix. Our phylogenetic analysis recovers *H*. *piceanus* as the sister taxon to *Glasbius*, which is consistent with our morphologic comparisons. *H*. *piceanus* represents the oldest member of the Glasbiidae. A regression equation for predicting body mass of dentally conservative metatherians that utilizes the length of m1 estimates the mass of *H*. *piceanus* at 855–1170 g, which is comparable in mass to today’s muskrat (*Ondatra zibethicus*) and large relative to other Late Cretaceous pediomyoids. Based upon its molar morphology, specifically the low inflated cusps, low height differential between the trigonid and talonid, and near-bunodont morphology, *H*. *piceanus* is interpreted as an omnivore with a plant-dominated diet.

## Introduction

The Metatheria (marsupials and their closest fossil relatives) comprise some 330 extant species in 7 orders, the great majority of which inhabit the Southern Hemisphere [1, 2]. However, the clade appears to have originated in the Northern Hemisphere during Early Cretaceous time [3]. By the end of the Cretaceous, metatherians had dispersed across Europe, Asia, and North America and were more diverse and abundant than their eutherian contemporaries [2, 4]. Most Late Cretaceous metatherian species are represented almost exclusively by isolated teeth and jaws recovered from fossil localities in the US Western Interior.

**Fig 1:**
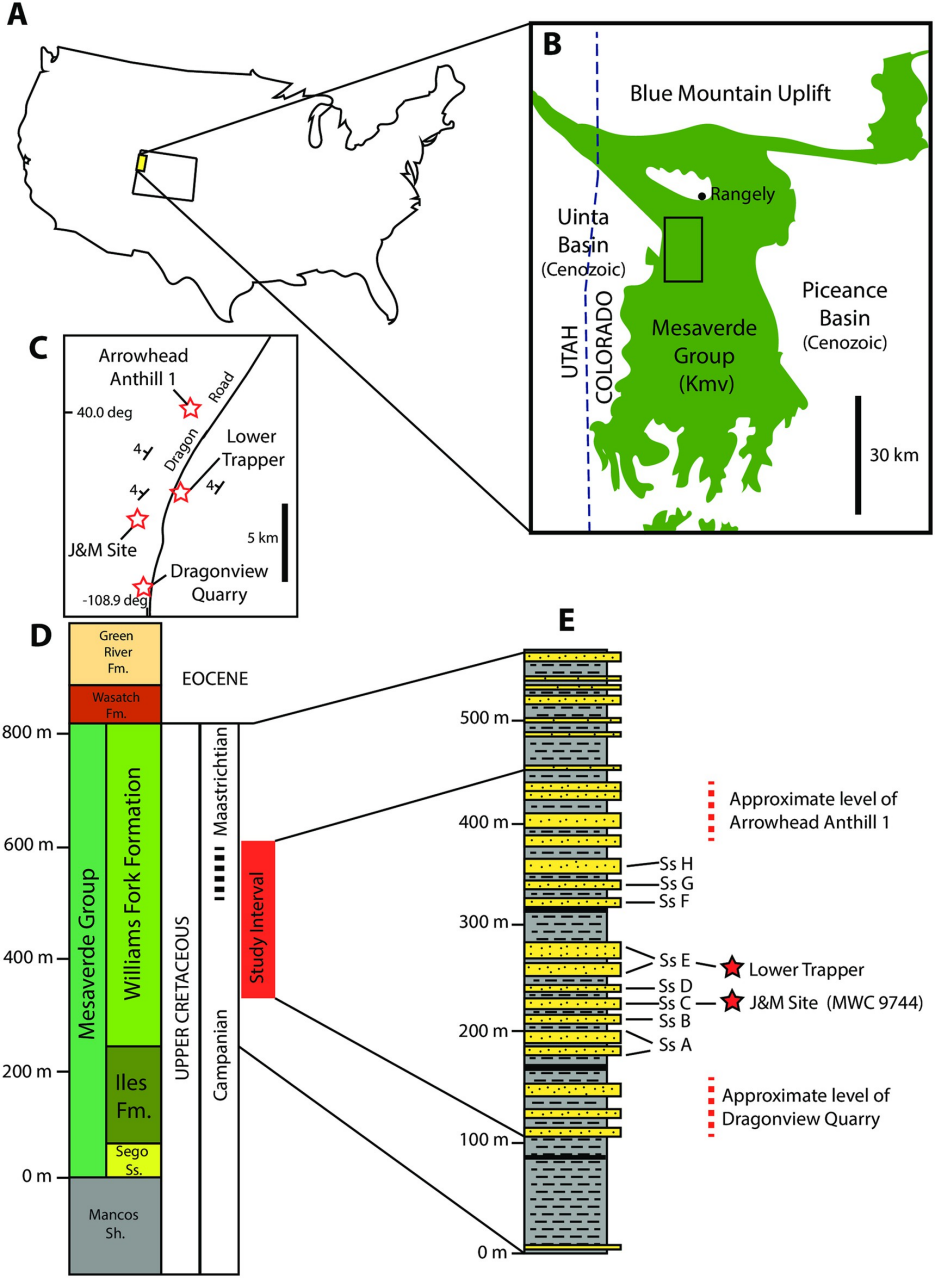
Geographic and stratigraphic location of the Williams Fork Formation study area and the new metatherian. (A) General location in western United States, with study area in northwestern Colorado marked by yellow box. (B) Enlargement showing exposures of Mesaverde Group (including Williams Fork Formation) in the Douglas Creek Arch (green) between the Uinta Basin to the west, Piceance Basin to the east, and Blue Mountain uplift to the north; smaller black rectangle identifies study area of the new metatherian; outcrop distribution from Tweto [16]. (C) Locations of the four localities that yielded the new metatherian (red stars) southwest of Rangely, Colorado; strikes and dips from Noll [19] [Unpublished]. (D) Regional stratigraphy in the study area showing the thickness, age, and neighboring units of the Williams Fork Formation and Mesaverde Group. (E) Stratigraphic section of the Williams Fork Formation showing Sandstones A–H and stratigraphic occurrences of the four specimens of the new metatherian (by locality). D and E based on data in Noll [19] [Unpublished].

Here, we describe a new genus of a relatively large metatherian (by Late Cretaceous standards) from Upper Cretaceous strata of the Williams Fork Formation on the Douglas Creek Arch, between the Uinta and Piceance Creek Basins in northwestern Colorado (Fig 1). The Williams Fork Formation comprises fluvio-deltaic and shallow marine deposits of late Campanian to early Maastrichtian (74–70 Ma) age [5, 6] that preserve a diverse vertebrate assemblage including dinosaurs, giant crocodilians, lizards, turtles, fishes, and mammals [7–12]. Among the dinosaurs, fossils of a chasmosaurine tentatively identified as *Pentaceratops* [8, 9, 13] as well as hadrosaurs and theropods are documented [10, 14]. Although not as well studied, the mammalian fossils recovered from the Williams Fork Formation are comprised largely of isolated teeth of multituberculates and metatherians, the latter of which include *Alphadon*, *Turgidodon*, *Eodelphis*, *Aenigmadelphis*, and tentatively *Leptalestes* [8, 11]. Prior to our study, only one mammalian dentary fragment, referred to the multituberculate *Meniscoessus collomensis*, had been documented from the Williams Fork Formation [15].

The fossils of the new metatherian described below were discovered on field expeditions to the Williams Fork Formation led by J. David Archibald (San Diego State University, CA) in the mid to late 1980s (JRF was a crew member in 1989), and more recent fieldwork (in the 2000s) by our team. Isolated molars of the new metatherian were initially referred to *Aquiladelphis incus* [8] and subsequently to *Glasbius piceanus* [17]. The discovery in 2018 of a dentary fragment containing m1–m3 (Museums of Western Colorado specimen 9744), the most complete therian fossil recovered to date from the Williams Fork Formation, helps refine the systematic relationships of the new genus which is neither *Aquiladelphis* nor *Glasbius*. We place the new taxon in phylogenetic context, and in light of its large size relative to many Late Cretaceous metatherians, we provide insight into its paleobiology.

## Geologic setting

The Upper Cretaceous Williams Fork Formation is exposed in the northwestern part of Colorado and is within the “Mesaverde Group” of this area (Fig 1); the latter unit appears to be slightly younger than the type Mesaverde Group near Durango, Colorado [18]. The Williams Fork Formation is late Campanian to earliest Maastrichtian in age at about 74–70 Ma [5, 6, 8, 19, 20] and it contains up to ~1000 m of various sediments representing diverse nonmarine and nearshore paleoenvironments, including floodplains, river channels, and swamps, plus deltaic, lagoon, beach, tidal channel, and shallow marine settings [21]. The marine units are more common in the eastern, type section outcrop areas and have produced some bivalves and foraminiferans [22].

The Williams Fork Formation overlies the Iles Formation and underlying Sego Sandstone, both also of the Mesaverde Group, and unconformably underlies the Eocene-aged Wasatch Formation (Fig 1). Regionally, the Williams Fork Formation is approximately chronostratigraphically equivalent to the Farrer and Tuscher formations in northeastern Utah, in part to the Pierre Shale and Fox Hills Sandstone farther east in Colorado [23–25] and in part the Kaiparowits Formation of southern Utah [26]. The unit is also approximately the same age as the Almond and Mesaverde formations in Wyoming, and the Hunter Canyon Formation in Colorado [6, 19, 25, 27]. Across western North America, the Williams Fork Formation appears to be roughly equivalent to the Bearpaw and Horseshoe Canyon formations of Alberta and Montana, the upper Aguja and lowermost Javelina formations of Texas, and the Kirtland Formation of New Mexico [10, 14, 28]. The Williams Fork Formation is likely slightly younger than the Judith River and Two Medicine formations of Montana [28]. Numerical ages suggest the Williams Fork Formation was deposited approximately ~74.5–72 Ma [28], or ~75.1–70.8 Ma [25], based largely on interpolation from ammonite zones above and below the Williams Fork Formation and correlative strata. Ages of a few ashes within the Williams Fork seem to align with these age ranges [6].

In an unpublished master’s thesis, Noll [19] [Unpublished] correlated a series of channel sandstones in the Williams Fork Formation in the study area, focusing on a series of eight channel sandstones (Sandstones A–H) that occupy approximately 200–225 m of section in the middle of the formation (Fig 1). These were correlated through mapping, measured sections, and fence diagrams, and Noll’s [19] [Unpublished] stratigraphic work is used to tie in at least two of the mammal localities studied here.

### History of collecting

The Williams Fork Formation contains plant macrofossils, pollen, freshwater mollusks, and a diverse vertebrate fauna based on locally abundant but mostly fragmentary specimens. Early reports of vertebrates include Lucas and Kihm [29], Lucas and Rasmussen [30], and Lillegraven [15]; most of the early work in the formation was carried out by crews led by J. David Archibald in the 1980s [7]. Among the better-preserved dinosaur specimens collected from the formation during this period were an incomplete chasmosaurine ceratopsian skull (SDMNH 43470), previously identified as either *Pentaceratops* [8, 13] or as Chasmosaurinae indet. [9], and a partial but unidentified skeleton of a hadrosaur (SDMNH 38229) collected in 1989 [8, 31]. A recently collected hadrosaur from just east of our study area was identified as a kritosaurin [32]. Other vertebrate taxa are also represented by isolated bones and teeth, including: indeterminate tyrannosaurids [8, 33]; several types of small theropods (all based on teeth); at least 15 species of mammals (these reflecting a mixed fauna of Judithian and Lancian taxa); the guitarfish *Myledaphus*; the gar *Lepisosteus*; salamanders and frogs; several lizards; a champsosaur, the crocodyliforms *Leidyosuchus* and/or *Brachychampsa*; and the turtles *Adocus*, *Aspideretoides*, and an indeterminate baenid [7, 14, 15, 34, 35]. Exploration by our group starting in 2009 has yielded additional specimens of many of these, as well as taxa new to the Williams Fork Formation, including: horsetail, angiosperm, and conifer plants; the giant amiid *Melvius*; the chondrichthyans *Lonchidion griffisi*, *Chiloscyllium*, *Cantioscylum markaguntensis*, *Cristomylus* and *Pseudomyledaphus*; the lizards *Peneteius* and *Leptochamops*; the turtle? *Denazinemys*, an oviraptorosaur [11, 12] and the mammal described herein.

Limited paleofaunal analyses of the Williams Fork Formation have concentrated on the dinosaurs and/or the mammals [8], and little has been produced regarding the other vertebrates [10, 11]. The Williams Fork Formation is like other regional deposits of approximately the same age, such as the Mesaverde Formation in Wyoming [36] and the Horseshoe Canyon Formation in Alberta [37], in being dominated in abundance and diversity by aquatic and semi-aquatic taxa [14].

### Localities with the new metatherian

The metatherian described below has been identified at four localities in the Williams Fork Formation. The type locality is Arrowhead Anthill #1 (UCM Loc. 66018); the most complete specimen is from the J&M site (MWC L-2012-13); and isolated teeth are also known from the Dragonview Quarry (UCM Loc. 93025) and the Lower Trapper site (UCM Loc. 86003).

#### Arrowhead Anthill #1 (UCM Loc. 86018)

The type locality Arrowhead Anthill #1 is a screen-washed anthill locality that is within meters of, and appears to be from the same layer as, a productive quarry site coined the Arrowhead Quarry (UCM Loc. 86017) that has produced fossils of turtles, crocodilians, and hadrosaurs, among others. There are several major sandstone bodies between these sites and the stratigraphically lower occurrences of the mammal described below, but the exact stratigraphic level of the Arrowhead sites has not been precisely determined. Based on the nearby Arrowhead Quarry, the Arrowhead Anthill #1 site seems to be eroding from a thin, medium-grained sandstone with abundant carbonaceous material at its base. This sandstone contains abundant bones and bone fragments in the quarry and seems to provide the source matrix for the material on the slope and in the anthills. The paleofauna of the Arrowhead sites includes: chondrichthyans; gars; an amphibian; turtles; a lizard; crocodylians; hadrosaurids; theropods such as dromaeosaurids, *Saurornitholestes*, and *Troodon*; and mammals such as *Turgidodon*, *Alphadon*, *Cimolomys*, and *Meniscoessus* [8].

#### J&M Site (MWC Loc. L-2012-13)

Museums of Western Colorado (MWC) 9744, a dentary fragment containing m1–m3 of the new metatherian, was recovered from the J&M Site, which was discovered in 2012 and has been worked intermittently since then. The site consists of a medium-grained sandstone scour lens within the base of Sandstone C of Noll [19] [Unpublished], in approximately the middle part of the Williams Fork Formation. The sandstone lens is approximately 5 m across and 50 cm deep at its thickest [10, 11], although Sandstone C is a 5–10 m thick unit that can be traced for many kilometers [19]. The sandstone lens of the J&M Site is coarser than most of the rest of Sandstone C, with a basal mud pebble- and bone conglomerate that contains many bones and teeth in random orientations; it also contains an abundance of coalified wood remains. Turtle shell fragments are particularly apparent in the outcrop. The site has produced many fossil taxa, including diverse chondrichthyans, actinopterygians including amiids and gars, several types of turtles, the polyglyphanodont lizard *Peneteius*, an apparent alligatoroid (cf. *Brachychampsa*), a large neosuchian, dinosaurs and several mammals [10–12].

#### Dragonview Quarry (UCM Loc. 93025)

This quarry is in an interbedded fine-grained sandstone and mudstone interval of approximately 3 m thickness that is approximately 100 m stratigraphically below Sandstone C. This locality has produced a diverse paleofauna including: gars; the chondrichthyans *Myledaphus* and *Myliobatis*; an amphibian; turtles; crocodylians; hadrosaurs; ceratopsians; the theropods *Saurornitholestes*, *Dromaeosaurus*, *Richardoestesia*, *Troodon*, and a tyrannosaurid; and mammals such as *Alphadon*, *Cimolodon*, and *Meniscoessus* [8].

#### Lower Trapper site (UCM Loc. 86003)

The Lower Trapper site is in a fine- to medium-grained sandstone within Sandstone E [19], making it ~60 m stratigraphically higher than the jaw locality at the J&M Site. The Lower Trapper site contains gars, *Myledaphus*, an amphibian, turtles, crocodylians, lizards, and theropods.

## Material and methods

### Specimen recovery

The University of Colorado Museum of Natural History (UCM) specimens are isolated teeth described originally in an unpublished master’s thesis by Diem SD [8] [Unpublished] that were collected by surface picking and screen-washing of anthills between 1984 and 1990 by crews from San Diego State University (SDSU) led by J. David Archibald. Museums of Western Colorado (MWC) 9744, a left dentary fragment with partial m1, m2–m3, is from the J & M Site (L-2012-13) and is on loan to JE from the MWC. Discovery of MWC 9744 took place in the lab in 2018 when MWC volunteer Tom Lawrence prepared the jaw fragment out of a fossiliferous sandstone block that had been removed from the site in 2016. The jaw fragment was not visible when the block was collected from the J & M Site, although other specimens such as turtle shell fragments were, indicating the jaw fragment was broken before burial. This is in line with the site’s taphonomy in that most preserved elements are somewhat fragmented, likely during fluvial transport. All necessary permits were obtained for the described study, which complied with relevant regulations. The sandstone block containing MWC 9744 was collected on Bureau of Land Management (BLM) Colorado Permit COC 77180 issued to JF. UCM specimens 57491, 57350, and 57354 were collected on BLM Colorado Permit C-41684 issued to J. David Archibald (San Diego State University, CA). All the localities are on land managed by the BLM south of Rangely in Rio Blanco County, northwestern Colorado. Detailed locality data are on file at the UCM and MWC (see also S1 Table).

### Dental nomenclature, measurements, and taxonomy

Studies agree that the therian ancestor of eutherians and metatherians had seven or eight postcanine teeth, including four or five premolars and three molars [2, 38]. O’Leary et al. [38] hypothesized that metatherians lost the permanent ultimate premolar (P5/p5) and retained the deciduous ultimate premolars DP5/dp5, which have traditionally been identified as M1/m1 in metatherian dentitions [39]. It was further postulated [38] that both metatherians and eutherians lost P3/p3 during evolution and consequently the metatherian postcanine formula contains: P1/p1, P2/p2, P4/p4, DP5/dp5, M1/m1, M2/m2, and M3/m3. Williamson et al. [2] accepted these homologies and identified the first tooth in the metatherian molar series as DP5/dp5, rather than M1/m1 as described in older literature. Although we do not disagree with these authors’ model for tooth homologies, we use the traditional metatherian tooth designations of P1/p1–P3/p3 and M1/m1–M4/m4 in our description of the metatherian from the Williams Fork Formation to facilitate comparison with the vast majority of metatherian taxa described in the literature.

The dental nomenclature used here follows Davis [40]. Dental measurements were obtained using a Zeiss Axio Zoom V16 high resolution zoom microscope with Axiocam 705 color camera and Zen Pro software. Measurement protocol follows Clemens [39]. Taxonomy follows Cohen et al. [17].

The phylogenetic analysis was conducted using TNT version 1.6 [41]. Detailed methods are provided prior to the presentation of the phylogenetic analysis. The character matrix was input and organized in Mesquite version 3.51 [42].

### Imaging

The specimens were scanned using micro computed tomography (CT) on a Zeiss Xradia 520 Versa^™^ X-ray microscope in the Biomechanics and Biomimetrics Laboratory at the University of Colorado Boulder. The voltage and power were 80–120 kV and 7–9 W, with higher values used on specimens encased in denser materials. The voxel sizes used depended upon the size of the specimen and ranged from 5.16–8.15 μm. Three-dimensional reconstructions were generated from micro-CT images using Dragonfly Pro software (Object Research Systems, ORS, Inc.) and conventional thresholding methods.

### Institutional abbreviations

**MWC**, Museums of Western Colorado, Dinosaur Journey Museum collections, Fruita, Colorado, USA; **UALVP**, Laboratory for Vertebrate Paleontology, Department of Biological Sciences, University of Alberta, Edmonton, Alberta, Canada; **UCM**, University of Colorado Museum of Natural History, Boulder, Colorado, USA; **UCMP**, University of California Museum of Paleontology, Berkeley, California, USA.

### Anatomical abbreviations and measurements

L, left tooth; R, right tooth; M/m, upper/lower molar; Wtri, width of trigonid; Wtal, width of talonid.

### Systematic paleontology

**Metatheria** Huxley, 1880

**Marsupialiformes** Vullo, Gheerbrant, Muizon, and Néraudeau, 2009

**Pediomyoidea** Simpson, 1927

**Remarks:** The Pediomyoidea Simpson, 1927 was raised to the rank of superfamily by Davis [40] to contain the families Pediomyidae, Glasbiidae, and Aquiladelphidae. A subsequent phylogenetic analysis by Williamson et al. [2] recovered the Aquilan genera *Aquiladelphis* and *Iqualadelphis* within the Pediomyidae, but *Glasbius* (the sole genus within Glasbiidae) was recovered as a more distantly-related metatherian allied with the South American Paleocene genus *Roberthoffstetteria*. When Eberle et al. [43] scored the Late Cretaceous Alaskan pediomyid *Unnuakomys* into the matrix of Williamson et al. [2], they recovered a clade containing *Glasbius* and *Roberthoffstetteria* as the sister group to the Pediomyidae. Other recent phylogenetic analyses [16, 44] also recovered *Glasbius* as the sister taxon to the Pediomyidae. We follow Cohen et al. [17] in using Pediomyoidea to encompass the families Pediomyidae and Glasbiidae. The oldest known definitive members of the Pediomyoidea are middle Turonian (ca. 92 Ma) in age [17].

***Heleocola*** gen. nov.

**Type and only known species:** *Heleocola piceanus* (Cohen et al. 2020).

**Diagnosis:** As for type species.

**Etymology:** ‘Heleo’, Greek word for marsh or swamp, in reference to the depositional environment of the Williams Fork Formation, and ‘cola’, Latin for dweller or inhabitant.

**Fig 2:**
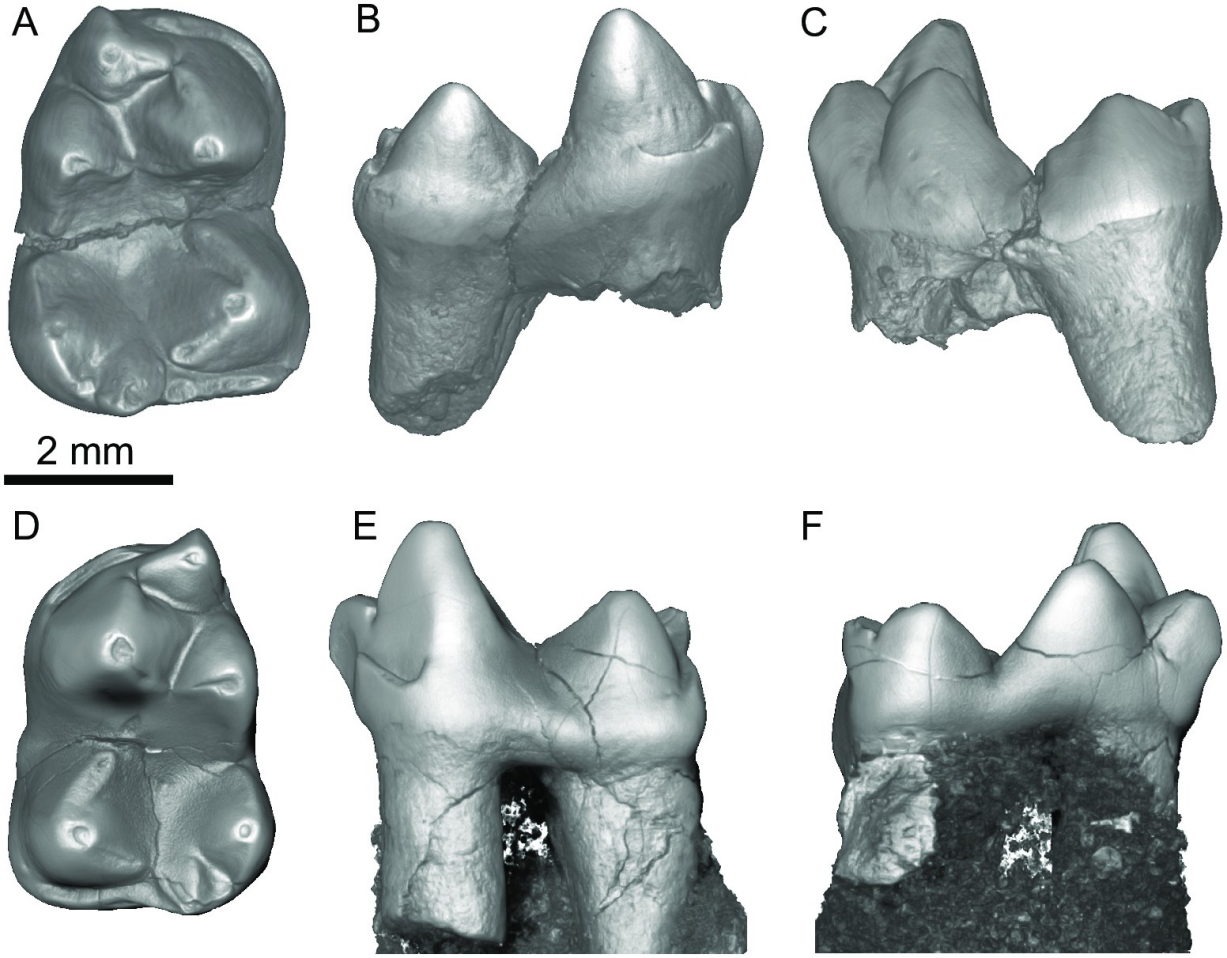
*Heleocola piceanus* from the Williams Fork Formation, Colorado. (A–C), Holotype UCM 57354, in occlusal (A), buccal (B), and lingual (C) aspects. (D–F) UCM 57491, in occlusal (D), buccal (E), and lingual (F) aspects.

**Fig 3:**
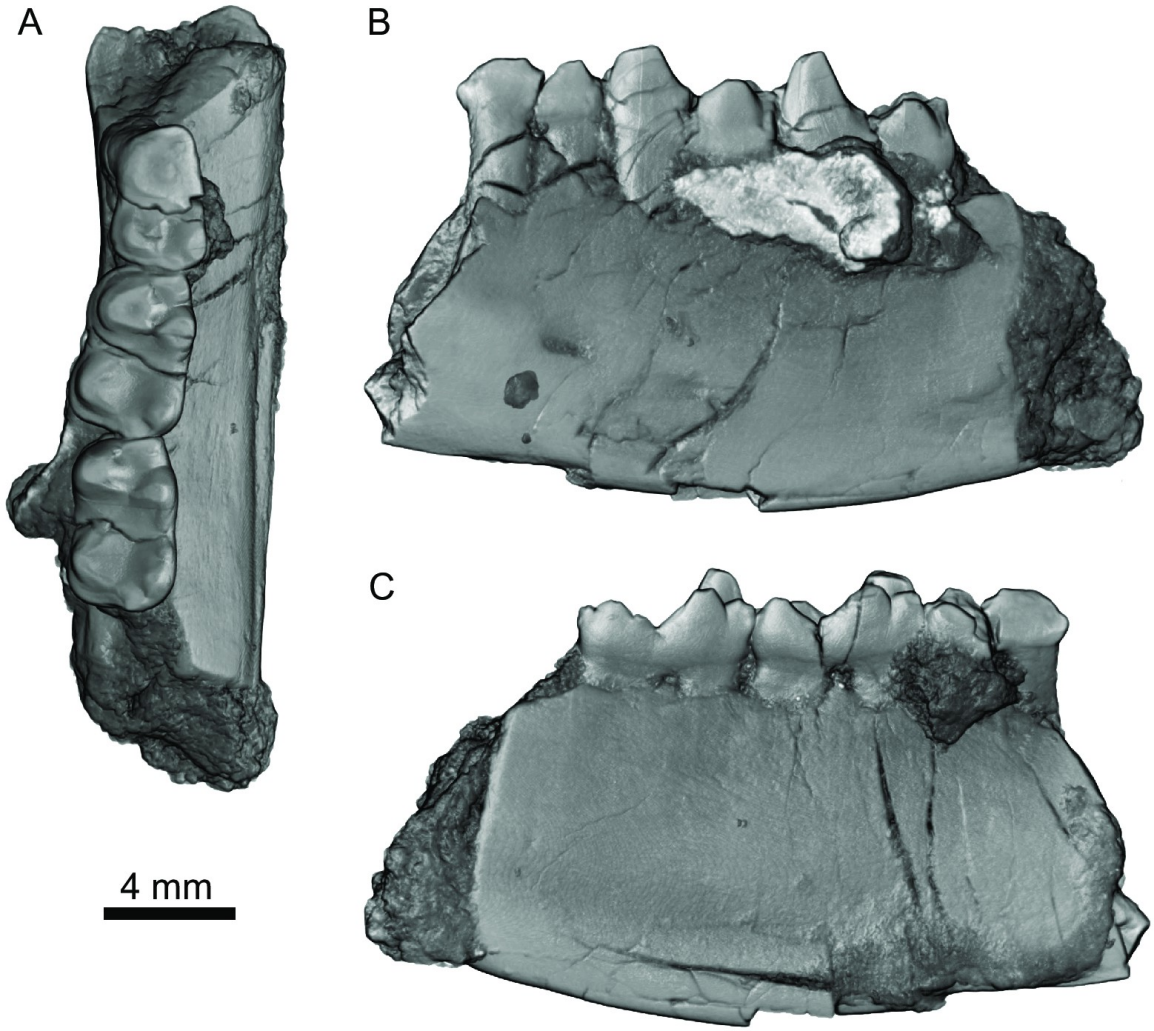
Left dentary fragment of *Heleocola piceanus* from *J&M Site (MWC Loc*. *L-2012-13)*, Williams Fork Formation, Colorado. (A–C), MWC 9744 in occlusal (A), buccal (B), and lingual (C) aspects.

**Fig 4:**
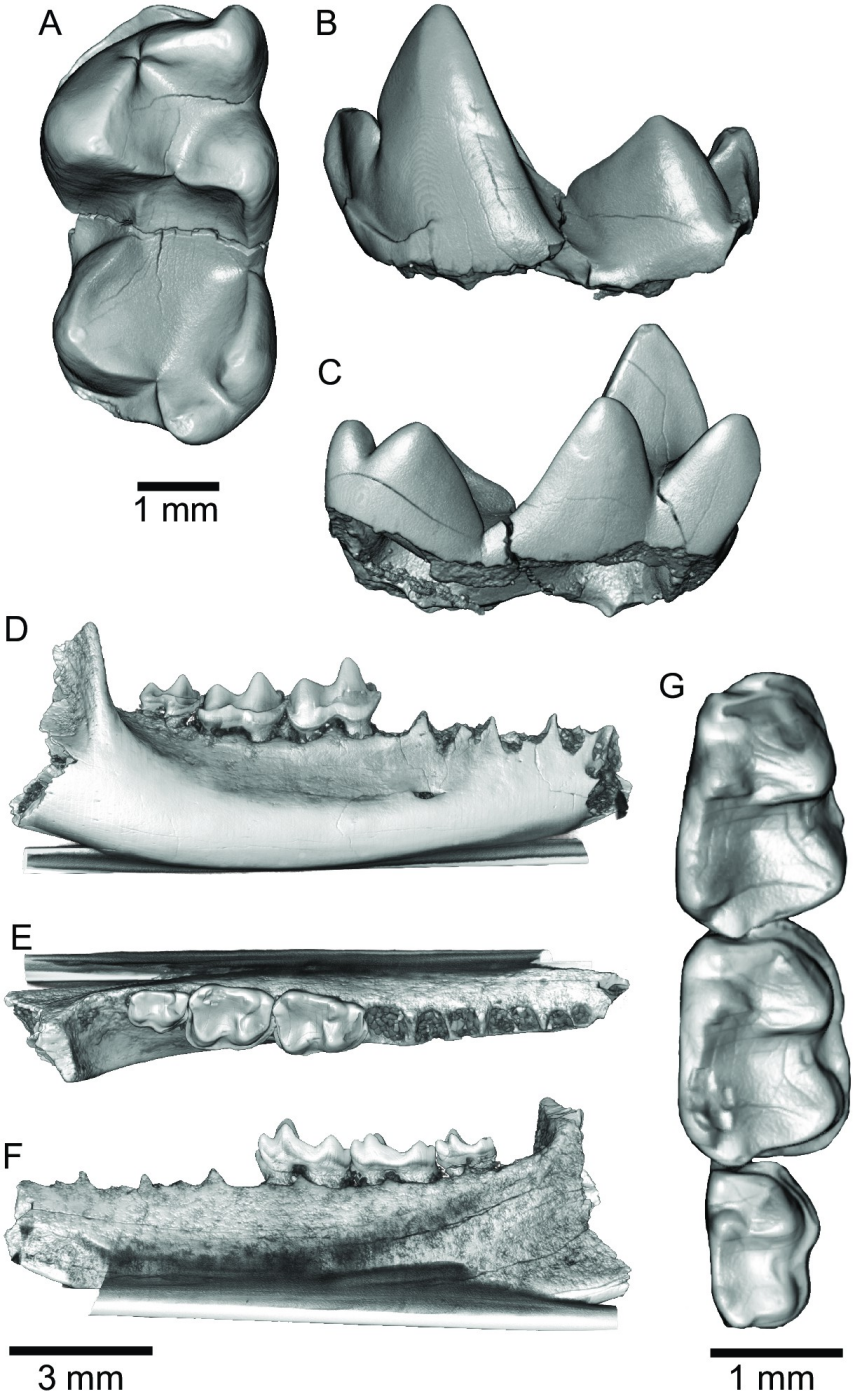
*Aquiladelphis incus* from Milk River Formation, Alberta, Canada and *Glasbius intricatus* from Hell Creek Formation, Montana, USA. (A–C) UALVP 29111, left mx in occlusal (A), buccal (B), and lingual (C) aspects. (D–G) UCM 53915, right dentary fragment with m2–m4 of *Glasbius intricatus* in buccal (D), occlusal (E, G), and lingual (F) aspects. Dentary has been digitally removed on (G).

*Heleocola piceanus* (Cohen et al. 2020) (Figs 2–4).

**Holotype and type locality:** UCM 57354, right m3, from Arrowhead Anthill #1 (UCM Loc. 86018), Williams Fork Formation, Douglas Creek Arch, Colorado (late Campanian—early Maastrichtian; ‘Edmontonian’ NALMA).

**Hypodigm:** MWC 9744, incomplete left dentary with partial m1, and m2–m3, from the J&M site (MWC locality L-2012-13); UCM 57350, worn Rm2 or m3 from Lower Trapper (UCM Loc. 86003); and UCM 57491, Lm1 from Dragonview Quarry (UCM Loc. 93025); all localities in Williams Fork Formation, Douglas Creek Arch, Colorado.

**Occurrence:** Known only from Upper Cretaceous (late Campanian—early Maastrichtian; ‘Edmontonian’ NALMA) strata of the Williams Fork Formation (Fig 1).

**Diagnosis:** Large pediomyoid metatherian whose lower molars are similar in size to *Aquiladelphis incus*; on m1–m3, trigonid is slightly taller than talonid, cusps are low and inflated, protoconid is tallest cusp, paraconid slightly lower than metaconid, cristid obliqua meets posterior wall of trigonid buccal to protocristid notch and approximately below the apex of protoconid; well developed pre- and postcingulids, but absence of a buccal cingulid, and talonid is wider than trigonid; m3 bears an entoconulid. Differs from *Aquiladelphis incus* in having transversely broader, lower molars with low, inflated cusps and a lower trigonid/talonid height differential, talonid wider than trigonid, a relatively longer talonid, metaconid taller than paraconid, and entoconulid on m3. Differs from *Glasbius* in much larger size, absence of a buccal cingulid, presence of entoconulid on m3, carnassiform notch on cristid obliqua and entocristid at juncture between talonid and trigonid, and a slightly more lingual attachment of the cristid obliqua to the distal trigonid wall.

**Description:** Our diagnosis and description of *Heleocola piceanus* is based on: two lower molars (UCM 57354 and UCM 57350; Fig 2) originally referred to *Aquiladelphis incus* [8] and subsequently to a new species of *Glasbius*, *G*. *piceanus* [17]; UCM 57491, a probable m1 initially referred to *Aquiladelphis incus* [8] (Fig 2); and a recently discovered dentary fragment (MWC 9744) with m1–m3 (Fig 3), all from the Williams Fork Formation. No upper dental elements are known. The holotype UCM 57354 and UCM 57350 were described in detail by Cohen et al. [17]. Below, we describe MWC 9744 and compare it with the UCM specimens. Measurements are provided in Table 1.

**Table 1:** Dental measurements (in mm) of lower molars of *Heleocola piceanus*.

Catalog No.	Position	Length	WTri	WTal
MWC 9744	m1	4.45	______	2.79^a^
	m2	5.20	3.29	3.38
	m3	5.30	3.26	3.37
UCM 57491	probable m1	4.59	2.74	3.14
UCM 57354	m3	5.03	3.21	3.65
UCM 57350	worn mx	4.42	3.32	3.54

^a^Specimen is worn and incomplete, precluding an accurate WTri measurement.

MWC 9744, a left dentary fragment, contains a worn, damaged m1 and complete m2–m3 that are slightly smaller in length, but wider than, a lower molar of early Campanian (Aquilan) *Aquiladelphis incus* (UALVP 5525) from the Milk River Formation, Alberta, Canada measured by Fox [46]. A mental foramen occurs ventral to the talonid of m1. A shallow Meckel’s Groove is present on the lingual side of the dentary, extending at least as far anteriorly as the m2. The m1 on MWC 9744 is smaller and transversely narrower than m2, the trigonid is worn flat and its cusps no longer discernible. Despite wear and damage on the talonid, the bases of the entoconid and hypoconid indicate relatively large cusps with a much smaller hypoconulid twinned and posterobuccal to the entoconid. On all three molars, the talonid is transversely broader than the trigonid, a characteristic of Pediomyoidea [40] that is especially pronounced in *Glasbius* [17]. The m2 and m3 on MWC 9744 are less worn than m1, although both contain transverse cracks between the trigonid and talonid, and the protoconid is broken off on m2. On m3, where all the cusps are intact, the protoconid is the tallest cusp. On both m2 and m3, the cusps are low and inflated, the paraconid is slightly lower than the metaconid, and its anteroposterior length is shorter than the metaconid in lingual view. The paraconid and metaconid are slightly appressed, with the paraconid positioned anterobuccally relative to the metaconid. The posterior wall of the trigonid dips steeply towards the talonid basin, and the cristid obliqua contacts the posterior wall of the trigonid buccal to the protocristid notch. A carnassiform notch occurs in the paracristid, and at the anterior ends of the entocristid and cristid obliqua where they contact the posterior wall of the trigonid. A narrow ridge extends posteriorly down the back of the metaconid, delimiting the anterior border of the entocristid notch. A pre- and postcingulid are present, both of which wrap around towards the buccal side, but do not form a buccal cingulid. The m3 on MWC 9744 bears a distinct entoconulid anterior to, and smaller than, the entoconid along the entocristid, whereas m2 lacks an entoconulid. This region of m1 is damaged and incomplete, so the presence or absence of an entoconulid is unknown. On all the molars, the entoconid is larger than the hypoconulid, and m2–m3 have a low trigonid/talonid height differential such that the entoconid is not much shorter than the metaconid. The entoconid is taller than the hypoconid, which characterizes the Pediomyidae [40]. The m2 and m3 on MWC 9744 are morphologically comparable to the holotype UCM 57354, with the main differences stemming from the relatively narrower talonid and lesser degree of inflation of the cusps on MWC 9744. UCM 57354 has an entoconulid-like structure on the entocristid, although it is not as large or distinct as on m3 of MWC 9744.

UCM 57491 is interpreted as a probable m1, based upon its similarity in size and morphology to the m1 on MWC 9744; its trigonid is considerably narrower than the talonid, which characterizes m1s of pediomyoids. The tooth is unworn, and the protoconid is the tallest cusp, while the much smaller paraconid projects anteriorly and is not appressed to the somewhat larger metaconid. As on the holotype UCM 57354 and the molars of MWC 9744, a carnassiform notch occurs in the paracristid and at the anterior end of the entocristid and cristid obliqua. UCM 57491 bears well developed pre- and postcingulids, but no buccal cingulid, and the entoconid is slightly lower than the metaconid. UCM 57491 is transversely narrower than UCM 57354, which is consistent with it being an m1.

### Remarks

The UCM specimens and MWC 9744 fall within the size range of lower molars of *Aquiladelphis incus*, which led Diem SD [8] [Unpublished] to refer the UCM specimens to this species. More recently, Cohen et al. [17] re-identified UCM 57350 and UCM 57354 as *Glasbius piceanus* and noted that the only character uniting the Williams Fork Formation teeth with *A*. *incus* was their large size. Our phylogenetic analysis, presented below and including the UCM specimens as well as MWC 9744, indicates that these teeth belong to neither *Aquiladelphis* nor *Glasbius*, but rather represent a new genus of large Late Cretaceous metatherian.

*Aquiladelphis incus* was described by Fox [45], along with the smaller *A*. *minor* and several other metatherians, from the early Campanian Milk River Formation in Alberta, Canada. Fox [45] described the lower molars (all isolated teeth) of *A*. *incus* as ‘large, broad, heavy teeth’ with low trigonid cusps, a buccally placed paraconid smaller than the metaconid, twinned entoconid and hypoconulid, and with heavy wear facets extending from the metaconid to the hypoconulid. Although comparable in size and having carnassiform notches similar to the Williams Fork Formation molars, the lower molars of *A*. *incus* (UALVP 29711; Fig 4) are morphologically quite different from the latter. In comparison to *Heleocola piceanus*, the lower molars of *A*. *incus* are not as broad and their cusps not nearly as inflated, the trigonid and talonid are subequal in width, the protoconid is noticeably taller and the paraconid and metaconid are nearly the same height, the hypoconulid is larger and posteriorly projecting, and there is a greater trigonid/talonid height differential.

Morphologically, *Heleocola piceanus* is more similar to the genus *Glasbius* than *Aquiladelphis*. *Glasbius* is known from two latest Cretaceous (Lancian) species, *G*. *intricatus* Clemens, 1966 (the type and better-known species; Fig 4) and the slightly larger *G*. *twitchelli* Archibald, 1982. Lower molars of *Heleocola piceanus* are approximately 2.5 times the size of lower molars of *G*. *intricatus* measured by Clemens [39] and about 2.3 times the size of those of *G*. *twitchelli* measured by Archibald [46]. Despite the significant size difference, lower molars of *Heleocola piceanus* share several characters with *Glasbius*, including having a talonid wider than the trigonid on m1–m3 and a lower trigonid/talonid height differential [17, this paper]. However, they differ from *Glasbius* in multiple characters, including absence of a buccal cingulid (which is well developed and often bearing small basal cusps in *Glasbius*), presence of a carnassiform notch at the anterior end of the cristid obliqua and entocristid, a slightly more lingual attachment of the cristid obliqua to the distal wall of the trigonid, and an entoconulid on m3. Additionally, on m3 of *Glasbius*, the paraconid and metaconid are more closely approximated than on m3 of MWC 9744.

### Phylogenetic analysis

Previous studies have referred isolated teeth of *Heleocola piceanus* to the genus *Glasbius* within the family Glasbiidae [17], or to *Aquiladelphis* within the Pediomyidae [8]. In light of the discovery of a dentary with m1–m3 (MWC 9744), we assessed the systematic relationships of *Heleocola piceanus* by scoring it into the taxon-character matrix of Williamson et al. [2], also used by Eberle et al. [43] in their analysis of the Alaskan pediomyid *Unnuakomys hutchisoni*. The analysis includes 97 taxa (four of which are non-metatherian) and 83 dental characters and uses the Jurassic basal eutherian *Juramaia sinensis* as the outgroup. As noted by Williamson et al. [2], the majority of Cretaceous and Paleogene metatherians are known only from teeth, so only dental characters were used in the analysis. We revised the scores of Williamson et al. [2] for three dental characters of *Glasbius twitchelli* (4, 74, and 76; see S1 Appendix), based upon the recent description of a relatively complete lower jaw from North Dakota [47]. *Heleocola piceanus* is known from lower molars and could be scored for only 20 of the 83 dental characters used by Williamson et al. [2]. The data matrix was analyzed using TNT version 1.6 [41], with multistate characters unordered. To ensure consistency with the analysis of Williamson et al. [2], the matrix was first analyzed under the ‘New Technology Search’ with Sectorial Search, Rachet, Drift, and Tree Fusing, finding the minimum-length tree 10 times. The 23 retained trees were then analyzed under the ‘Traditional Search’ with tree bisection reconnection (TBR) branch-swapping. Bremer support values were calculated using a pool of 30,000 suboptimal trees of up to 10 steps longer than the shortest trees.

## Results

**Fig 5:**
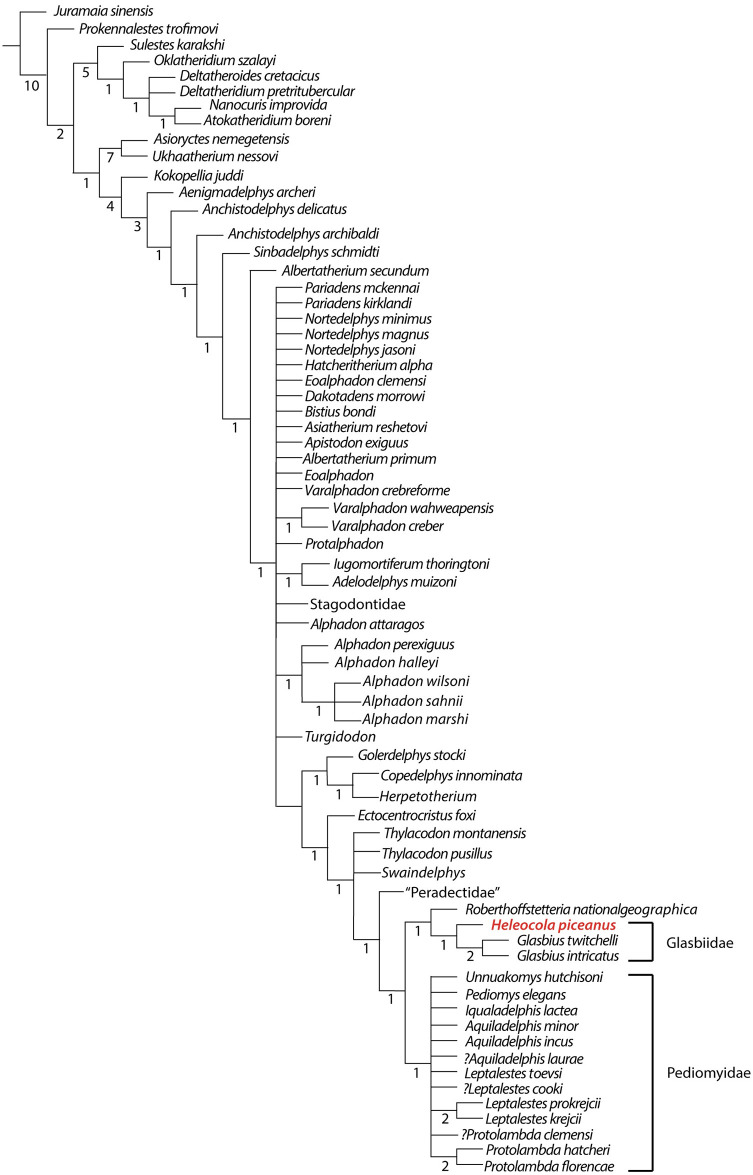
Simplified phylogeny of Metatheria based upon strict consensus of 491 trees of 534 steps (consistency index 0.197; retention index 0.700) in S2 Fig. Numbers at each node correspond to Bremer branch supports calculated from a pool of 30,000 suboptimal trees of up to 10 steps longer than the shortest trees. *Heleocola piceanus* (in red) is recovered as the sister taxon to *Glasbius*; this clade along with early Paleocene Bolivian polydolopimorph *Roberthoffstetteria nationalgeographica* form the sister group to the Pediomyidae.

The analysis resulted in 491 trees of 534 steps (consistency index 0.197; retention index 0.700). The low ensemble consistency index suggests considerable homoplasy in the dataset, probably a result of the large amounts of missing data for some taxa, including *Heleocola piceanus*, as well as the nearly 1:1 ratio of taxa to characters [2]. In the simplified strict consensus tree (Fig 5), on the whole the topology is similar to that of Williamson et al. [2], save for the inclusion of *Heleocola piceanus* which is recovered as the sister taxon to the clade containing *Glasbius* (the entire strict consensus tree can be found in S2 Fig). This sister group relationship is consistent with our morphologic comparisons (above) and the conclusion of Cohen et al. [17] that the isolated lower molars of *H*. *piceanus* are more similar to *Glasbius* than they are to *Aquiladelphis incus*, to which Diem SD [8] [Unpublished] had initially referred them. The clade containing *Heleocola* and *Glasbius* (Family Glasbiidae) is found to be the sister group to the early Paleocene Bolivian polydolopimorph *Roberthoffstetteria nationalgeographica*. All three genera have low, inflated cusps with a low trigonid/talonid height differential, the metaconid is taller and mesiodistally longer than the paraconid, and the entoconid is larger than the hypoconulid. Synapomorphies of the upper molars of *R*. *nationalgeographica* and *Glasbius* include the relative size of the stylar cusps and the large metaconule [48]. These authors also noted the incipient development of a supernumerary cusp between the metaconid and entoconid on lower molars of *R*. *nationalgeographica*, although not as distinct as that on m3 of MWC 9744. Williamson et al. [2] also recovered *R*. *nationalgeographica* as the sister to *Glasbius*, corroborating earlier studies [48–50] that suggested a close relationship between these taxa. Goin et al. [48] considered *Glasbius* a potential sister group to the Polydolopimorphia. Alternatively, and given the incompleteness of the fossils, this may be an example of convergent evolution.

A notable difference between our results and those of Williamson et al. [2] is that the clade containing *R*. *nationalgeographica*, *H*. *piceanus*, and *Glasbius* spp. is found to be the sister group of the Pediomyidae *sensu* Williamson *et al*. [2]. Eberle et al. [43] recovered the same result and identified several characters of the upper molars that unite *Glasbius* spp. + *Roberthoffstetteria* with the Pediomyidae. More pertinent to our study, lower molars of *H*. *piceanus*, *Glasbius* spp., *R*. *nationalgeographica*, and the Pediomyidae share the following characters: the talonid is either subequal to, or wider than, the trigonid, and the cristid obliqua contacts the posterior trigonid wall buccal to the protocristid notch. However, Bremer support for our tree topology is low, in part due to large amounts of missing data. Therefore, our results should be considered preliminary, until more material of *Heleocola piceanus* and other poorly represented taxa is recovered.

## Discussion

Fossil mammals from the Williams Fork Formation are rare and almost entirely represented by isolated teeth. Our report is the first jaw fragment of a therian from this rock unit. Our referral of *Heleocola piceanus* to the Glasbiidae based on the phylogenetic analysis extends the temporal range of the family from the Lancian back into the “Edmontonian” and westward into western Colorado. Prior to our study, the Glasbiidae included only *Glasbius*, which occurs in Lancian faunas in Wyoming, Montana, and Saskatchewan, Canada to the north [40] and in New Mexico at its southernmost range extent [51].

Lower molars of *Heleocola piceanus* are large relative to other pediomyoids, similar in size to those of *Aquiladelphis incus*, but over twice the size of Lancian *Glasbius intricatus* and *Pediomys elegans* measured by Clemens [39], and over three times the size of lower molars of contemporaneous (“Edmontonian”) pediomyid *Unnuakomys hutchisoni* from northern Alaska measured by Eberle et al. [43]. Its large teeth beg the question, how large of a mammal was *Heleocola piceanus*? A commonly used proxy for estimating body mass of fossil mammals relies on dental dimensions, specifically the size of the first lower molar [52]. For dentally conservative metatherians including opossums, the first molar is highly correlated with body size, although there is a strong relationship between body size and tooth size in the more posterior molars too [53]. We estimated the body mass of *Heleocola piceanus* by incorporating the length of the m1s (n = 2) into the regression equations for didelphids and dasyurids of Gordon [53]. Body mass for *Heleocola piceanus* is estimated to have been 855–1170 g, which is similar in mass to today’s muskrat (*Ondatra zibethicus*; 910–1250 g) [54]. *H*. *piceanus* weighed about half that of the largest known Late Cretaceous metatherian, the stagodontid *Didelphodon vorax* whose estimated body mass ranged from 2.4–5.2 kg [44].

Whereas the localities containing *Heleocola* are to the west of most other occurrences of glasbiids, the Williams Fork Formation was deposited as a prograding fluvio-deltaic suite of deposits [5, 6], with abundant carbonaceous beds. The overall faunal assemblage, which contains terrestrial, freshwater, estuarine and marine taxa including numerous sharks and rays, suggests *Heleocola* lived near a low-lying, swampy to deltaic environment near the margin of the Western Interior Seaway [11, 12].

### Paleobiological interpretations

The lower molars of *Heleocola piceanus* exhibit several notches, including between the metaconid and the entoconid, within the cristid obliqua, and within the protocristid and centrocristid. Carnassiform notches are often associated with carnivory or insectivory, so species that exhibit this morphology are typically interpreted as incorporating carnivory into their overall diet. Additionally, species with carnivorous diets typically exhibit sharp crests and shearing blades on their molars, but this is not the case in *H*. *piceanus*. Beyond the lack of sharp crests, *H*. *piceanus* molars exhibit a number of characteristics suggesting a diet more in line with plant-dominated omnivory. The cusps of *H*. *piceanus* are inflated, with a low height differential between the trigonid and talonid, a near-bunodont morphology typically associated with the grinding and crushing of plant materials [55, 56]. In general, the molar morphology largely agrees with a plant-dominated omnivorous diet, with blunt crests, inflated cusps, and a low trigonid/talonid height differential, with the exception of the presence of carnassiform notches. There are a few hypotheses that could explain the presence of the notches associated with multiple traits typically associated with plant-dominated omnivory. The notches could reflect an ancestral trait, where the ancestor to *H*. *piceanus* incorporated more carnivory or insectivory into the diet, or the notches could be unrelated to these dietary modes. These notches are typically referred to as carnassiform notches due to their superficial similarity to the notches found in the carnassials of members of the Carnivora (and other carnivorous mammalian groups). This association has led to the interpretation that the presence of these notches indicates carnivory or insectivory [57–59]. The function of the notch has been described as a mechanism to capture and hold food during shearing, thereby improving the functional efficiency of the shearing crests [59, 60]. So functionally, these notches are not necessarily unique to carnivores and insectivores, but could be found, to some degree, in any mammal that utilizes shearing forces, including carnivory, insectivory, and folivory. Indeed, the presence of notches, referred to as either a lingual notch or a talonid notch, has been documented in several primate taxa, where diets are more aligned with frugivory or plant-dominated omnivory [61, 62]. Therefore, the presence of a notch should not necessarily lead to a dietary inference towards carnivory or insectivory, as frugivorous and folivorous taxa can also exhibit this morphology. Taking all the morphology together, we interpret *H*. *piceanus* as a plant-dominated omnivore. This inferred diet of *H*. *piceanus* agrees with the results for other members of Glasbiidae from Brannick et al. [63], where *Glasbius intricatus* and *Glasbius twitchelli* were recovered primarily as plant-dominated omnivores. Its larger size implies a greater need for highly nutritious foods, so *H*. *piceanus* may have incorporated insects and/or small vertebrates into its diet along with roots, fruits, and nuts.

## Supporting information

S1 Table Coordinates for fossil localities from which *Heleocola piceanus* was recovered in the Late Cretaceous Williams Fork Formation in western Colorado. (PDF)

S1 Appendix List of taxa and characters used in phylogenetic analysis. Save for the addition of *Heleocola piceanus* and revised scores for characters 4, 74, and 76 for *Glasbius twitchelli* based upon description of new fossils of this species [47], we did not modify the list of taxa, characters, or the scores for the taxa from Eberle et al. [43] who, in turn, incorporated Alaskan metatherian *Unnuakomys hutchisoni* into the taxon-character matrix of Williamson et al. [2] and modified these authors’ Character 26. Note: Williamson et al. [2] use the postcanine dental homologies hypothesized by O’Leary et al. [38] in their character descriptions and identify the first tooth in the molar series in Metatheria as homologous to the DP5/dp5 of Eutheria.
(PDF)

S1 Fig Taxon-character matrix (Nexus file). Note: there are 97 taxa and 83 dental characters, of which *Heleocola piceanus* (known only from lower molars) could be scored for 20 of them.
(DOCX)

S2 Fig Strict consensus of 491 trees of 534 steps calculating using TNT version 1.6 (CI = 0.197; RI = 0.701). *Heleocola piceanus* (in red) is recovered as the sister taxon to *Glasbius*. Numbers at nodes correspond to Bremer branch supports calculated from a pool of 30,000 suboptimal trees of up to 10 steps longer than the shortest trees.
(TIF)
