# Supplementary material for: A new Late Cretaceous metatherian from the Williams Fork Formation, Colorado
Source: PLoS One. 2024 Oct 23;19(10):e0310948. doi: 10.1371/journal.pone.0310948 (PMC11498682; doi:10.1371/journal.pone.0310948)
Supplement: S1 Table — (PDF) [file pone.0310948.s001.pdf]

Table of GPS coordinates for the fossil localities from which *Heleocola piceanus* was recovered in the Late Cretaceous Williams Fork Formation in western Colorado. As per the BLM Permanent Instruction Memorandum (PIM) 2022-009 (https://www.blm.gov/policy/pim2022-009), the location of paleontological resources may be provided to the public with a precision that is no greater than 0.1 decimal degrees latitude/longitude.

Locality Number and Name	GPS coordinates (latitude; longitude)
MWC L-2012-013 J&M site	39.9; -108.9
UCM Loc. 86018 Arrowhead #1	40.0; -108.9
UCM Loc. 93025 Dragonview Quarry	39.9; -108.9
UCM Loc. 86003 Lower Trapper Site	39.9; -108.9
