# Supplementary material for: A new Late Cretaceous metatherian from the Williams Fork Formation, Colorado
Source: PLoS One. 2024 Oct 23;19(10):e0310948. doi: 10.1371/journal.pone.0310948 (PMC11498682; doi:10.1371/journal.pone.0310948)
Supplement: S1 Appendix — Save for the addition of Heleocola piceanus and revised scores for characters 4, 74, and 76 for Glasbius twitchelli based upon description of new fossils of this species [47], we did not modify the list of taxa, characters, or the scores for the taxa from Eberle et al. [43] who, in turn, incorporated Alaskan metatherian Unnuakomys hutchisoni into the taxon-character matrix of Williamson et al. [2] and modified these authors’ Character 26. Note: Williamson et al. [2] use the postcanine dental homologies hypothesized by O’Leary et al. [38] in their character descriptions and identify the first tooth in the molar series in Metatheria as homologous to the DP5/dp5 of Eutheria. (PDF) [file pone.0310948.s002.pdf]

Taxa

Adelodelphys muizoni (Cifelli, 2004)

Aenigmadelphys archeri Cifelli and Johanson, 1994

Albertatherium primum Fox, 1971

Albertatherium secundum Johanson, 1995

Alphadon attaragos Lillegraven and McKenna, 1986

Alphadon halleyi Sahni, 1972

Alphadon marshi Simpson, 1927

Alphadon perexiguus Cifelli, 1994

Alphadon sahni Lillegraven and McKenna, 1986

Alphadon wilsoni Lillegraven, 1969

Anchistodelphys archibaldi Cifelli, 1990b

Anchistodelphys delicatus Cifelli, 1990a

Apistodon exiguus Fox, 1971

?Aquiladelphis laurae Eaton, 2006

Aquiladelphis incus Fox, 1971

Aquiladelphis minor Fox, 1971

Armintodelphys blacki Krishtalka and Stucky, 1983

Armintodelphys dawsoni Krishtalka and Stucky, 1983

Asiatherium reshetovi Trofimov and Szalay, 1993

Asioryctes nemegetensis Kielan-Jaworowska, 1975

Atokatheridium boreni Kielan-Jaworowska and Cifelli, 2001

Bistius bondi Clemens and Lillegraven, 1986

Copedelphys innominata (Simpson, 1928)

Dakotadens morrowi Eaton, 1993

Deltatheridium pretrituberculare Gregory and Simpson, 1926

Deltatheroides cretacicus Gregory and Simpson, 1926

Didelphodon coyi Fox and Naylor, 1986

Didelphodon vorax Marsh, 1889

Ectocentrocristus foxi Rigby and Wolberg, 1987

Eoalphadon clemensi (Eaton, 1993)

Eoalphadon lillegraveni (Eaton, 1993)

Edaphon woodburnei Eaton, 2009

Eodelphis browni Matthew, 1916

Eodelphis cutleri Woodward, 1916

Glasbius intricatus Clemens, 1966

Glasbius twitchelli Archibald, 1982

Golerdelphys stocki Williamson and Lofgren, 2014

Hatcheritherium alpha Case et al. 2005

Heleocola piceanus Eberle et al. (this paper)

Herpetotherium comstocki Cope, 1884

Herpetotherium edwardi Gazin, 1952

Herpetotherium fugax (Cope, 1873)

Herpetotherium knighti McGrew, 1959

Herpetotherium marsupium Troxell, 1923

Iqualadelphis lactea Fox, 1987

Iugomortiferum thoringtoni Cifelli, 1990b

Juramaia sinensis Luo et al. 2011

Kokopellia juddi Cifelli, 1993a

?Leptalestes cooki (Clemens, 1966)

Leptalestes krejcii (Clemens, 1966)

Leptalestes prokrejcii (Fox, 1979)

Leptalestes toevsi Hunter et al. 2010

Maastrichtidelphys meurismeti Martin et al. 2005

Mimoperadectes houdei Horovitz et al., 2009

Mimoperadectes labrus Bown and Rose, 1979

Nanocuris improvida Fox et al. 2007

Nortedelphys jasoni (Storer, 1991)

Nortedelphys magnus Case et al. 2005

Nortedelphys minimus Case et al. 2005

Oklatheridium szalayi Davis et al. 2008

Pariadens kirklandi Cifelli and Eaton, 1987

Pariadens mckennai Cifelli, 2004

Pediomys elegans (Marsh, 1889)

Peradectes californicus (Stock, 1936)

Peradectes chesteri (Gazin, 1952)

Peradectes elegans Matthew and Granger, 1921

Peradectes gulottai Rose, 2010

Peradectes coproxeches Williamson and Taylor, 2011

Peradectes minor Clemens, 2006

Peradectes protinnominatus McKenna, 1960

Prokennalestes trofimovi Kielan-Jaworowska and Dashzeveg, 1989

Protalphadon foxi Johanson, 1996b

Protalphadon lulli (Clemens, 1966)

?Protolambda clemensi Sahni, 1972

Protolambda florencae (Clemens, 1966)

Protolambda hatcheri Osborn, 1898

Pucadelphys andinus Marshall and Muizon, 1988

Roberthoffstetteria nationalgeographica Marshall et al. 1983

Sinbadelphys schmidti Cifelli, 2004

Sulestes karakshi Nessov, 1985

Swaindelphys cifellii Johanson, 1996a

Swaindelphys encinensis Williamson and Taylor, 2011

Swaindelphys johansoni Williamson and Taylor, 2011

Szalinia gracilis Muizon and Cifelli, 2001

Thylacodon pusillus Matthew and Granger, 1921

Thylacodon montana Williamson et al., 2012

Turgidodon lillegraveni Cifelli, 1990a

Turgidodon madseni Cifelli, 1990a

Turgidodon petiminis Storer, 1991

Turgidodon praesagus (Russell, 1952)

Turgidodon rhaister Clemens, 1966

Turgidodon russelli (Fox, 1979)

Ukhaatherium nessovi Novacek et al. 1997
Unnuakomys hutchisoni Eberle et al. 2019
Varalphadon creber (Fox, 1971)
Varalphadon crebreforme (Cifelli, 1990c)
Varalphadon wahweapensis (Cifelli, 1990b)

Characters

1. Lower incisor number: No more than 3. (0); 4 or more. (1). Modified from Horovitz & Sánchez-Villagra [1], character 151; Horovitz et al. [2], character 148; Rougier et al. [3, 4], character 42.

2. Lower incisor staggering: Not staggered. (0); Staggered (medially staggered position of root and alveolus of i2; i2 larger than i1, i3, or i4). (1). Condition described in Cifelli & de Muizon [5, 6], Hershkovitz [7, 8] and Marshall & de Muizon [9], but with the i2 being staggered rather than the i3 as originally described by Hershkovitz [7] as was proposed by Sánchez-Villagra et al. [10] and followed by Horovitz et al. [2], character 169; Rougier et al. [3. 4], character 43.

3. C: Double rooted. (0); Single rooted. (1). From Horovitz & Sánchez-Villagra [1], character 171; Horovitz et al. [2], character 168.

4. Number of lower premolars: Five. (0); Four or fewer. (1). Modified from Kielan-Jaworowska et al. [11], character 108; modified from [3, 4], character 1.

5. First upper premolar: Single rooted. (0); Double rooted (1).

6. First lower premolar: Oriented in line with jaw axis. (0); Oblique. (1). From Averianov et al. [12], character 43.

7. Deciduous precursors for ante-P4/p4 dentition: Present (0); Absent (1).

8. DP4 mesial stylar shelf: Narrow, but present. (0); Present as an ectocingulum (1); Absent. (2). Ordered.

9. DP4 parastylar lobe: Moderate in size and stylar cusp A is in line with paracone and metacone. (0); Expanded and stylar cusp A is positioned lingual to a line drawn from the paracone and metacone. (1).

10, DP4 stylar cusp B: Present (0); Absent (1).

11. DP4 stylar cusp C: Present. (0); Absent. (1).

12. DP4 stylar cusp C size: Smaller than stylar cusp D. (0); Larger than stylar cusp D. (1).

13. P4: Absent. (0); Small lingual bulge. (1); With an enlarged basin. (2). From Rougier et al. [3, 4], character 12. Ordered.

14. Number of roots on P4: Two. (0); Three. (1). From Rougier et al. [3, 4] character 13.

15. P5/p5 (permanent replacement tooth): present, (0); absent, deciduous tooth retained (1).

16. Inflation of P4/p4: Uninflated (main cusp of p4 is narrower than the trigonid of m1). (0); Somewhat inflated (the width of the main cusp of p4 is subequal to that of m1). (1); Markedly inflated (p4 is wider and longer than m1; P4 buccolingual width approaches that of M1). Lingual lobe or "accessory lobe" present. (2). Modified from Davis [13], character 56. Ordered.

17. Morphological features on the buccal cingulum or stylar shelf of the upper molars (excluding the parastyle and metastyle): Distinctive cingulum, without cuspules. (0); Individualized or even hypertrophied cuspules (1). Modified from Luo et al. [14], character 99.

18. Stylar cusp A (dP5–M2): Absent. (0); Present. (1).

19. Stylar cusp D: Absent. (0); Variably present. (1); Consistently present. (2). From Davis [13], character 10. Ordered.

20. Stylar cusps B, C, and D: Stylar cusp D present, C and B absent. (0); Stylar cusp D present, C and B present. (1); Stylar cusp D and C present, B absent. (2); Stylar cusp D and B present, C absent. (3). Modified after Johanson [15], character 6; Davis [13], character 6 and 8; Rougier et al. [3, 4], character 23; Vullo et al. [16], character 6.

21. Stylar cusp A (parastyle) position relative to stylar cusp B (stylocone): Lower than cusp B. (0); Closely approximated to cusp B. (1); Modified after Johanson [17], character 2.

22. Stylar cusp A size relative to stylar cusp B (penultimate molar): Equal to or larger than stylar cusp B. (0); Smaller than stylar cusp B. (1). From Rougier et al. [3, 4], character 20.

23. Stylar cusp B (stylocone) size: Small (less than half the size of the paracone). (0); Moderately developed (greater than about half the size of paracone, but smaller than paracone). (1); Large (subequal to paracone). (2). Modified after Johanson [17], character 3; Rougier et al. [3, 4], character 22; Davis [13], character 3. Ordered.

24. Position of stylar cusp B (stylocone) relative to paracone: Positioned nearly directly buccal to paracone. (0); Positioned mesiobuccal to paracone. (1). From Davis [13], character 5.

25. Stylar cusp C size: Small (present as a distinct cusp, but is equal to or smaller than stylar cusp A). (0); Well-developed (greater than stylar cusp A, but less than stylar cusp D). (1); Large (equal to or greater than stylar cusp B or D. (2). Modified after Davis [13], character 8; Vullo et al. [16], character 6. Ordered.

26. Presence of two stylar cusps in the cusp C position: Absent. (0); Variably present. (1); Consistently present. (2). Ordered. Modified from Williamson et al. [18], character 26.

27. Position of cusp C relative to the ectoflexus (dP5–M2): Positioned at, or mesial to, the deepest part of the ectoflexus. (0); Positioned distal to the deepest part of the ectoflexus. (1). Modified after Johanson [17], character 7; Davis [13], character 9.

28. Position of cusp C relative to the buccal edge of the stylar shelf (dP5–M2): Positioned along the buccal edge of the stylar shelf. (0); Positioned lingual to the buccal edge of the stylar shelf. (1). Modified after Johanson [17], character 8.

29. Relative size of stylar cusps C and D (M2): Stylar cusp C is smaller than D. (0); Stylar cusp C is equal to or larger than D. (1). Modified after Johanson [17], character 10.

30. Cusp C shape in buccal view: Conical. (0); Squared at its apex. (1). Modified after Johanson [17], character 11.

31. Stylar cusp D size (M2): Moderately developed. (0); Small. (1). Modified from Davis [13], character 11; Vullo et al. [16], character 7.

32. Position of Stylar cusp D relative to metacone: Positioned buccal to metacone. (0); Positioned mesiobuccal to metacone and closer to deepest part of ectoflexus. (1). From Davis [13], character 12.

33. Stylar cusp D shape at base: Broad and mesiodistally long at base. (0); Conical at base (1). From Davis [13], character 14.

34. Mesial portion of stylar shelf (parastylar lobe) (M2): Not reduced. (0); Somewhat reduced so that it is buccolingually narrower than metastylar lobe. (1); Greatly reduced so that just a rim remains buccal to paracone and stylar cusp A is nearly directly mesial to paracone. (2). From Davis [13], characters 1 and 15 which are here combined. Ordered.

35. Preparacrista strength: Well-developed. (0); Weakly-developed or absent. (1). From Davis [13], character 16.

36. Preparacrista orientation: Preparacrista runs to a position below apex of cusp B (lingual side of cusp B) or to the mesiolingual face of cusp B. (0); Preparacrista runs to position mesial to the apex of cusp B or toward stylar cusp A if stylar cusp B is absent. (1). Modified from Davis [13], character 17.

37. Presence of carnassial notch along preparacrista: Present. (0); Absent. (1). From Davis [13], character 18.

38. Presence of carnassial notch along postmetacrista: Present. (0); Absent. (1). From Davis [13], character 20.

39. Ectoflexus depth (M2): Deep (embayment equal to or greater than 10% of total width of tooth). (0); Shallow (embayment less than 10% of total width of tooth). (1). From Davis [13], character 21.

40. Ectoflexus across molar series (dP5–M2): Increases in depth distally through the molar series. (0); Little or no change in depth of ectoflexus along the molar series. (1). Modified after Johanson [17], character 15; Davis [13], character 22.

41. Relative height of the paracone and metacone (dP5-M2). Paracone is taller than the metacone. (0); Paracone and metacone are subequal in height. (1); Metacone is taller than the paracone. (2). Modified from Johanson [17], character 16; Davis [13], character 23. Approximately equivalent to Horovitz & Sánchez-Villagra [1], character 155; Horovitz et al. [2], character 152; Vullo et al. [16], character 10; Rougier et al. [3, 4], character 27. Ordered.

42. Relative size of paracone and metacone in buccal view (as measured mesiodistally; dP5–M2): Paracone is longer than the metacone. (0); Paracone and metacone are of equal size. (1); Metacone is longer than the paracone. (2). Modified from Johanson [17], character 17; Davis [13], character 24. Approximately equivalent to Horovitz & Sánchez-Villagra [1], character 155;

Horovitz *et al.* [2], character 152; Vullo et al. [16], character 10; Rougier et al. [3, 4], character 27. Ordered.

43. Paracone and metacone shape (dP5–M2): Uninflated and unrounded. (0); The paracone is inflated and rounded compared to the metacone. (1); Both the paracone and metacone are inflated and rounded. (2). Modified from Davis [13], character 25.

44. The shape of the buccal faces of the paracone and metacone (dP5–M2): Flat or concave. (0); Buccal face of paracone is convex (rounded); buccal face of metacone is flat. (1); Convex or rounded. (2). Modified from Davis [13], character 26; Rougier et al. [3, 4], character 29.

45. The paracone and metacone relative separation at base (dP5–M2): Share a portion of their bases. (0); Entirely separate at their bases. (1). From Davis [13], character 27; Vullo et al. [16], character 11; Rougier et al. [3, 4], character 30.

46. Centrocrista morphology (refers to location of the deepest point of the centrocrista relative to an imaginary line between the apices of the paracone and metacone): Straight (equivalent to a U-shaped ectoloph). (0); Deflected buccally so that it is V-shaped (equivalent to a W-shaped ectoloph). (1); Invades stylar shelf as disconnected crests. (2). Modified from Johanson ([17], character 23; Horovitz & Sánchez-Villagra [1], character 156; Horovitz et al. [2]; Davis [13], character 29; Kielen-Jaworowska et al. [11], character 82; and after Case et al. (2005); Rougier et al. [3, 4], character 31.

47. Paraconule and metaconule size: Small and weakly-developed (dP5–M2). (0); Large and strongly-developed. (1). Modified from Johanson [17], character 24; Davis [13], character 30; Vullo et al. [16], character 8.

48. Position of paraconule relative to protocone and paracone: Relatively closer to protocone or midway between protocone and paracone. (0); Relatively closer to paracone. (1). Modified from Davis [13], character 31.

49. Strength and morphology of the internal cristae: Well-developed. (0); Weakly-developed or absent. (1). From Davis [13], character 32; modified from Rougier et al. [3, 4], character 35.

50. Protocone height relative to paracone/metacone (whichever is taller): Shorter than paracone/metacone. (0); Approaching height of paracone/metacone (over half the height). (1). Modified from Davis [13], character 33; Rougier et al. [3, 4], character 38.

51. Protocone position: Positioned mesiodistally midway between paracone and metacone. (0); Positioned mesial to the midway point between paracone and metacone. (1).

52. Protocone procumbency: Absent. (0); Present (1). From Rougier et al. [3, 4], character 37.

53. Protocone basal distal expansion (M1-2): Unexpanded. Protocone is V-shaped. (0); Expanded so that part of the protocone is somewhat lobe-shaped or "squared off." (1). Modified from Davis [13], character 35.

54. Pre- and postcingula: Absent. (0); Precingulum present, postcingulum absent. (1); Pre- and postcingula present. (2). Modified from Johanson [17], character 28; Davis [13], character 36; Vullo et al. [16], character 15.

55. Placement of paraconule, protocone, and metaconule: Not aligned in a row. (0); Aligned in a row. (1). From discussion by Goin et al. [20] and Case et al. [19]).

56. Preprotocrista: Terminates lingual of base of paracone. (0); Joins preparaconular crista and extends buccally past base of paracone (presence of double rank prevallum/postvallid shearing). (1). From Cifelli [21], Rougier et al. [3, 4], character 33.

57. Postprotocrista: Absent. (0); Present. (1).

58. Postprotocrista length (dP5–M2): Extends from protocone or merges with postmetaconule crista only to base of metacone. (0); Extends buccally to wrap around the distal side of metacone, but does not extend to distobuccal corner of tooth (all molars but last). (1); Extends beyond buccal base of metacone to near buccal margin of tooth. (2). Modified from Cifelli [21], Davis [13], character 39 and 41, Rougier et al. [3, 4]; Vullo et al. [16], character 9. Ordered.

59. Postmetacrista orientation (M2): Wide Line drawn between paracone and metacone forms approximately a right angle with postmetacrista. (0); Line drawn between paracone and metacone forms an obtuse angle (greater than about 100 degrees) with postmetacrista. (1).

60. Relative transverse widths of the trigonid and talonid (M1-M2): Trigonid is wider than talonid. (0); Talonid and trigonid are of subequal width. (1); Talonid is significantly wider than trigonid. (2). Modified from Johanson [17], character 32; Davis [13], character 42; Horovitz & Sánchez-Villagra [1], character 158; Horowitz et al. [2], character 155; Rougier et al. [3, 4], character 50; Vullo et al. [16], character 30. Ordered.

61. Relative lengths of trigonid and talonid (dP5-M2): long (trigonid length/talonid length is 1.0 or less). (0); Talonid short (trigonid length/talonid length is greater than 1.0). (1).

62. Relative mesiodistal lengths of paraconid and metaconids in lingual view (dp5–2): The paraconid is longer than the metaconid. (0); The two cusps are relatively equal in length. (1); The metaconid is longer than paraconid. (2). From Davis [13], character 45. Ordered.

63. Relative heights of paraconid and metaconid: Paraconid is taller than metaconid. (0); Two cusps are subequal in height. (1); Metaconid is taller than paraconid. (2). From Davis [13], character 44 and modified from Vullo et al.[16], character 22; Rougier et al. [3, 4], character 59. Ordered.

64. Mesiolingual face of the paraconid: Not strongly keeled. (0); Strongly keeled, sometimes with basal cusp. (1).

65. Position of paraconid relative to metaconid: Positioned buccally relative to metaconid. (0); Positioned more lingually, such that the paraconid, metaconid and entoconid, if all present, line up mesiodistally. (1). From Davis [13], character 46 and modified from Vullo et al. [16], character 22.

66. Position of paraconid relative to metaconid (m1–3): Paraconid projects mesially. (0); Paraconid appressed to the metaconid. (1). Modified from Kielan-Jaworowska et al. [11] and discussed by Fox & Naylor [22]. This also essentially includes Kielan-Jaworowska et al. [11], character 72 (angle between the paracristid and protocristid), because, with the paraconid closely appressed to the metaconid, the angle between the paracristid and the protocristid is low.

67. Angle of trigonid (measured from entoconid to protoconid, with metaconid as vertex; all molars but first): Obtuse (greater than 95 degrees). (0); Approaches 90 degrees (equal to or less than 95 degrees). (1). From Davis [13], character 48.

68. Molar talonid: Narrow, lacking entoconid. (0); With at least three cusps, including entoconid (1).

69. Size of entoconid relative to hypoconulid (dp5–m2): Smaller than hypoconulid. (0); Comparable in size to hypoconulid. (1); Larger than hypoconulid. (2). From Davis [13], character 49; modified from Vullo et al. [16], character 32. Ordered.

70. Height of entoconid relative to metaconid (m1–m2): Less than or equal to 0.30 height of metaconid. (0); Greater than 0.30 height of metaconid. (1). Modified from Davis [13], character 49; Rougier et al. [3, 4], character 54.

71. Shape of entoconid (dp5–m2): Buccolingually compressed and blade-like. (0); Conical. (1). Springer et al. [23], character 20; Ladevèze & de Muizon [24], character 65.

72. Position of hypoconulid relative to entoconid (dp5–m2): Hypoconulid and entoconid are not twinned or twinning is weak, hypoconulid is near tooth midline. (0); Hypoconulid and entoconid

are strongly twinned, but hypoconulid is buccal to a position that is distal to entoconid. (1); Hypoconulid and entoconid are twinned and hypoconulid is positioned on the lingual margin of tooth, distal to entoconid. (2). Modified from Davis [13], character 50; Vullo et al. [16], character 32; Rougier et al. [3, 4], character 52. Ordered.

73. Postcingulid (dp5-m2): Absent. (0); Present. (1).

74. Postcingulid (m3): Absent. (0); Present. (1).

75. Accessory cusp adjacent to entoconid along entocristid (entoconulid; dp5–m2): Absent. (0); Present. (1). From Davis [13], character 51.

76. m3 entoconid: Same relative size as in preceding molars. (0); Reduced compared to preceding molars. (1).

77. Presence of talonid ectocingulid (as extension of postcingulid): Absent. (0); Present. (1). From Davis [13], character 52.

78. Ventral extent of talonid portion of crown (in buccal view): Extends ventrally to level of trigonid or slightly past level of trigonid (ventral margin of crown is horizontal or slopes gently distally). (0); Significantly expanded such that the ventral margin of crown slopes steeply distally. (1). Modified from Davis [13], character 53.

79. Cristid obliqua (dp5–m2): Incomplete; postmetacristid present. (0); Complete. (1). Modified from Rougier et al. [3, 4], character 51.

80. Cristid obliqua (dp5–m2): Meets the distal trigonid wall at a point lingual or ventral to the protocristid notch. (0); Meets the distal trigonid wall buccal to the protocristid notch, approximately below apex of protoconid. (1); Meets the distal trigonid wall buccal to the protocristid notch near buccal margin of tooth. (2). Ordered. Modified from Davis [13], characters 53 and 55; Horovitz & Sánchez-Villagra [1], character 160; Horovitz et al. [2],

character 157; Johanson [17], character 35; Vullo et al. [16], character 27; Rougier et al. [3, 4], character 51. Ordered.

81. m3 cristid obliqua: Meets the distal trigonid wall at a point lingual or ventral to the protocristid notch. (0); Meets the distal trigonid wall buccal to the protocristid notch. (1).

82. Estimated mass (ln g) based on tooth measurements (after Gordon 2003): Small (equal to or less than 4). (0); Medium (greater than 4, equal to or less than 6). (1); Large (greater than 6). (2). Modified from Davis [13], character 57. Ordered.

83. m3 molar size: m3 is small relative to dp5 (m3 Length/dp5 Length less than 0.8. (0); m3 is not small or large relative to dp5 (m3L/dp5 L equal to or greater than 0.8, less than 1.5). (1); m3 is large relative to dp5 (m3 L/dp5 L greater than 1.5). (2). Modified from Rougier et al. [3, 4], character 61. Ordered.

References

1. Horovitz I, Sánchez-Villagra MR. A morphological analysis of marsupial mammal higher-level phylogenetic relationships. Cladistics. 2003;19:181–112.
2. Horovitz I, Martin T, Bloch JI, Ladevèze S, Kurz C, Sánchez-Villagra MR. Cranial Anatomy of the earliest Marsupials and the origin of opossums. PLOS ONE. 2009:4:e8278.
3. Rougier GW, Wible JR, Novacek MJ. Implications of *Deltatheridium* specimens for early marsupial history. Nature. 1998;396:459–463.
4. Rougier GW, Wible JR, Novacek MJ. New Specimen of *Deltatheroides cretacicus* (Metatheria, Deltatheroida) from the Late Cretaceous of Mongolia. Bulletin of Carnegie Museum of Natural History. 2004:36: 245–266.
5. Cifelli RL, de Muizon C. Dentition and jaw of *Kokopellia juddi*, a primitive marsupial or near-marsupial from the medial Cretaceous. Journal of Mammalian Evolution. 1997;4:241–258.

6. Cifelli RL, de Muizon C. Marsupial mammal from the Upper Cretaceous North Horn Formation, central Utah. Journal of Paleontology. 1998;72:532–537.
7. Hershkovitz P. The staggered marsupial lower third Incisor (I3). Géobios. 1982;6:191–200.
8. Hershkovitz P. The staggered marsupial third lower incisor: Hallmark of cohort Didelphimorphia, and description of a new genus and species with staggered i3 from the Albian (Lower Cretaceous) of Texas. Bonner Zoologische Beitrage. 1995;45:153–169.
9. Marshall LG, de Muizon C. The dawn of the age of mammals in South America. National Geographic Research. 1988;4:23–55.
10. Sánchez-Villagra M, Ladeveze S, Horovitz I, Macrini TE, Martin, JE, Morre-Fay S et al. 2007. Exceptionally preserved North American Paleogene metatherians: Adaptations and discovery of a major gap in the opossum fossil record. Biological Letters. 2007;3:318–322.
11. Kielan-Jaworowska Z, Cifelli, RL, Luo, Z-X. 2004. Mammals from the Age of Dinosaurs: Structure, Relationships, and Paleobiology. New York: Columbia University Press; 2004.
12. Averianov AO, Archibald JD. Ekdale EG. New material of the Late Cretaceous deltatheroidan mammal *Sulestes* from Uzbekistan and phylogenetic reassessment of the metatherian-eutherian dichotomy. Journal of Systematic Palaeontology. 2010; 8:301–330.
13. Davis BM. A revision of "pediomyid" marsupials from the Late Cretaceous of North America. Acta Palaeontologica Polonica. 2007:52:217–256.
14. Luo Z-X, Yuan C-X, Meng Q-J, Ji Q. A Jurassic eutherian mammal and divergence of marsupials and placentals. Nature. 2011; 476:442–445.
15. Johanson Z. New marsupial from the Fort Union Formation, Swain Quarry, Wyoming. Journal of Paleontology. 1996a;70:1023–1031.
16. Vullo R, Gheerbrant E., de Muizon C, Néraudeau D. The oldest modern therian mammal from Europe and its bearing on stem marsupial paleobiogeography. Proceedings of the National Academy of Science (USA). 2009;106:19910–19915.
17. Johanson Z. Revision of the Late Cretaceous North American marsupial genus *Alphadon*. Palaeontographica Abteilung A. 1996b;242:127–184.

18. Williamson TE, Brusatte SL, Wilson GP. The origin and early evolution of metatherian mammals: the Cretaceous record. ZooKeys. 2014;465:1–76.
19. Case JA, Goin FJ, Woodburne MO. "South American" marsupials from the Late Cretaceous of North America and the origin of marsupial cohorts. Journal of Mammalian Evolution. 2005;11:223–255.
20. Goin FJ, Candela AM, De Muizon C de. The affinities of *Roberthoffstetteria nationalgeographica* (Marsupialia) and the origin of the polydolopine molar pattern. Journal of Vertebrate Paleontology. 2003;23:869–876.
21. Cifelli RL. Theria of metatherian-eutherian grade and the origin of marsupials. In Szalay FS, Novacek MJ, McKenna MC, editors. Mammal Phylogeny: Placentals, New York: Springer-Verlag; 1993. p. 205–215.
22. Fox RC, Naylor BG. Stagodontid marsupials from the Late Cretaceous of Canada and their systematic and functional implications. Acta Palaentologica Polonica. 2006;51: 13–36.
23. Springer MS, Kirsch JAW, Case JA. The chronicle of marsupial evolution. in Givnish TJ, Sytsma KJ, editors. Molecular Evolution and Adaptative Radiation, New York: Cambridge University Press; 1997. p. 129–161.
24. Ladevèze S., de Muizon C. The auditory region of early Paleocene Pucadelphydae (Mammalia, Metatheria) from Tiupampa, Bolivia, with phylogenetic implications. Palaeontology. 2007;50:1123–1154.
