# Supplementary material for: A new Late Cretaceous metatherian from the Williams Fork Formation, Colorado
Source: PLoS One. 2024 Oct 23;19(10):e0310948. doi: 10.1371/journal.pone.0310948 (PMC11498682; doi:10.1371/journal.pone.0310948)
Supplement: S1 Fig — Note: there are 97 taxa and 83 dental characters, of which Heleocola piceanus (known only from lower molars) could be scored for 20 of them. (DOCX) [file pone.0310948.s003.docx]

#NEXUS

BEGIN DATA;

DIMENSIONS NTAX=97 NCHAR=83;

FORMAT DATATYPE = STANDARD RESPECTCASE GAP = - MISSING = ? SYMBOLS = " 0 1 2 3";

MATRIX

Juramaia_sinensis 10011???????001011231110------10000011010002000011000001120?1?10?0?110?000??0????01

Prokennalestes_trofimovi ???1?0??????101011231110------1000001100000200101100000111100220010000100000001000?

Ukhaatherium_nessovi 1?1110??????211011231011------00000000110002001001000001101001100111101001000010011

Asioryctes_nemegetensis 1001?0??????211011231011------00000000110002001001000001101002200111101001000010011

Adelodelphys_muizoni ????????????????110-0110---------000??000002001001010001110???2????10?011?0?0?1?00?

Aenigmadelphys_archeri ???????????????011230010------00000000100000000011110001110100100001110111000010?01

Albertatherium_primum ????????????????1121011010001000100001001100101001110001110??????????????????????11

Albertatherium_secundum ????????????????11230110------001000000?1100101001110001110??????????????????????1?

Alphadon_attaragos ???????????????01121011000010000000001??000010110111000111?11220100111011?0?0010?0?

Alphadon_halleyi ???0???????????01121011010(0 1)1(0 1)0001000010012021011011?0001110112101001110111000010001

Alphadon_marshi ????????????????1121011010011000100001001202101101110001110111101001110111000010011

Alphadon_perexiguus ????????????????1121011010111000100001?0120210110111000111011??0100111011?0?0010?01

Alphadon_sahnii ????????????????112101101001100010000100120210110111000111011110100111011?0?0010?11

Alphadon_wilsoni ???????????????0112101101001100010000101120210110111000111011110100111011?0?0010?11

Anchistodelphys_archibaldi ????????????????111(1 3)01100000000000001001110000100111000111011210000111011?0?0010?01

Anchistodelphys_delicatus ????????????????110-????---------000?1??000000?????1????1101121000011?011?0?0010?0?

Apistodon_exiguus ????????????????1121011110101000110001100002100001110001110??????????????????????0?

?Aquiladelphis_laurae ????????????????11211?0112001000110-?????1??101?0111110111???????????????????????2?

Aquiladelphis_incus ???????????????2112110012110100011011011012210111111120111011200110121011?0?001112?

Aquiladelphis_minor ????????????????11211?0120111000120-?01?011110111111100111??02?0100121011?0?0011?11

Armintodelphys_blacki ???????????????????????????????????????????????????????????00(0 1)101001001101010011101

Armintodelphys_dawsoni ????????????????1121111020?010000001110?110010001111000110000110100100111?0?0011?01

Asiatherium_reshetovi ??1010??????00?01123-000------00001--1001102001001100201110111201001111111000011111

Atokatheridium_boreni ????????????????100-0100---------0101000000200001011000110001000100000--00-?000--00

Bistius_bondi ????????????????11210?1010101010101011??0021101001110201100??????????????????????2?

Copedelphys_innominata ????????????????11210110100000000000110032001110011110011011022010012012110?0011?11

Dakotadens_morrowi ????????????????112(1 3)0110101000000011101?(0 1)(0 1)01100011110001100111101001211100000011?1?

Deltatheridium_pretrituberculare 101000??????00?0100-11?0---------00000000002000-1010000110101001100000100000000--10

Deltatheroides_cretacicus ??10?0??????00?0100-1100---------00???000002000010110001101??????????????????????10

Didelphodon_coyi 00?011??????00?21123?120------00001010??2222101101110001?00100011111210111100011?22

Didelphodon_vorax 0?1011?(1 2)010000?211231120------00001110012222101101110001100100011111210111100012122

Ectocentrocristus_foxi ????????????????11210110200000?0000011??220011101111101110?????11001?????1??001?01?

Eoalphadon_clemensi ???????????????0112101101011(0 1)0001000010000021010111100011101122010011101110?0010?01

Eoalphadon_lillegraveni ????????????????112(1 3)011000010000100001000002101011110001110111(1 2)010011?011?0?0010?1?

Eoalphadon_woodburnei ????????????????11210110000100001000010?00021010111100011101112010012?011?0?0010?1?

Eodelphis_browni 0010?1??????00?211230120------00000010??222210110111000110?10001111121?11?1?0011?22

Eodelphis_cutleri ???01???????00?211230120------0000001001222210110111000110010001111121011?1?0011?22

Glasbius_intricatus ???????????????01121111000(0 1)10000101111112222101111111101120212201001210111001012110

Glasbius_twitchelli ???1?1?011000??01121111000010000101111112222101111111101120212201001210110001012110

Hatcheritherium_alpha ????????????????112101100011?0?01?1011??220012110111020111???????????????????????1?

Golerdelphys_stocki ????????????????11210110100010001000111?320011001111000110110220101121011?0?0011?1?

Herpetotherium_comstocki ????????????????112101?0000000111001111?3200111011111001101102201001211211001011111

Herpetotherium_edwardi ????????????????11210100100000011001111?3200111001111001101102201001211211000011111

Herpetotherium_fugax 10101??????????01121010010000001100111103200111001111001101102201001211210000011011

Herpetotherium_knighti ????????????????112(1 3)010010101001100111113200111001111001101102201001211211000011111

Herpetotherium_marsupium ???????20100???0112101?0(1 2)010100110011100320011100111100110?102201001201211001011?11

Iqualadelphis_lactea ????????????????11231101------00010101002100001101110101120??????????????????????01

Iugomortiferum_thoringtoni ????????????????100-011010111010001011??002200001101000111?112201001110111010010001

Kokopellia_juddi 10?0?1?????????0110-0010---------00000000002000001100001100101100001100011000010?01

?Leptalestes_cooki ???????????????01120-0-1------10021-1100120100110111110112010220100121011?0?0011?1?

Leptalestes_krejcii ???????10100???01120-0-1------10021-1100220100000111100112010120100111011?0?0011?0?

Leptalestes_prokrejcii ???????????????01120-0-1------10021-1100110100000111100112010120100?11011?1?0011?0?

Leptalestes_toevsi ????????????????11231001------00011-110???0?1001111110011201?1201001????1???0011?01

Maastrichtidelphys_meurismeti ????????????????11211000200010?00?0011??220011101111000110???????????????????????0?

Mimoperadectes_houdei ??1?1???????00??112(1 3)111000000000000111002222100011110001100??????????????????????11

Mimoperadectes_labrus ???0???????????0112111100000000000011111122210001111000110001000100110111001(0 1)011011

Nanocuris_improvida ????????????????10230100------000?10111?0002000-1010000110101000100000000001000--20

Nortedelphys_jasoni ???????????????01121011010010000100001001200111101110001110112201001210111000010?11

Nortedelphys_magnus ???????????????01121011010010000100001002200111101110001110112201001210111010010011

Nortedelphys_minimus ????????????????11210110100100001000?10?1200111101110001110??????????????????????0?

Oklatheridium_szalayi ????????????????110-0100---------000000101020010101000011010?00010????????????0-?0?

Pariadens_kirklandi ????????????????112101101001000010111???11221010111000011??100011001210110000011012

Pariadens_mckennai ???????????????????????????????????????????????????????????100111001???11?0?0010012

Pediomys_elegans ???0???????????0112210-121101000121111002211101111111101120202201111210111100112111

Peradectes_californicus ???????1001-???011231110------0000101111220010001111000110??1?2010011011110?0011?01

Peradectes_chesteri ????????????????110-1110---------0001100110010001111000110?1122010011011110?0011101

Peradectes_elegans ???01??????????0112111100000000000001110220010001111000110011220100110111100(0 1)011101

Peradectes_gulottai ????????????00?011231110------00000111002200100011110001100??????????????????????01

Peradectes_coproxeches ????????????????1121111010(0 1)00000000111002200101011110001100112201001200111000011101

Peradectes_minor ????????????????1121111010000000000111002200101011110001100112201001100111010011001

Peradectes_protinnominatus ???????????????011211110000000000001111011001000111100011001122010011011110?1011?01

Protalphadon_foxi ????????????????112(1 3)01100000(0 1)01010001100(0 1)100111001110001100??????????????????????1?

Protalphadon_lulli ???0?1??????????112(1 3)01100000001010001100010010100111000110?10110100121011?0?1010?0?

?Protolambda_clemensi ???????????????11120-0--------01021-1?1010111011?111100112011220110121011?000011101

Protolambda_florencae ???????????????11120-0--------00021-1?1111111011?111100112020110100121011?000011111

Protolambda_hatcheri ???????(1 2)0100???11120-0--------00021-1?1112111011?1111001120201101001210111000012111

Pucadelphys_andinus 111011??????00?01121101010001000100000002100111001110001100102201001101111010011101

Roberthoffstetteria_nationalgeographica ????1???????00??11211110100100001001111122221210111110111201022001012111000?0011112

Sinbadelphys_schmidti ????????????????110-0110---------000000011020010011100011101021010011?0111000010001

Sulestes_karakshi 011011?20101?0??11??1110---------00(0 1)0001000200001000000110101000100100100000000--01

Swaindelphys_cifellii ????????????????1121111010100000000111102200111011110001100102201001110111000011111

Swaindelphys_encinensis ????????????????1121111010000000000111102200111011110001100102201001110111010011111

Swaindelphys_johansoni ???????21000????1121111010000000000111102200111011110001100102201001110111010011111

Szalinia_gracilis 1110????????00?0112(1 3)101000000000100001002200110011110001100002201001001110010011101

Thylacodon_pusillus ???????????????01121111010000000000111102200101011110001100102201001210111000011111

Thylacodon_montanensis ???????211?????01121111010000000000111002200101011110001100102201001210111010011111

Turgidodon_lillegraveni ???????????????0112101201001010000100??11122101101110001110??????????????????????1?

Turgidodon_madseni ??????????????01112101201001110000000?11112210110111000111010220100121011?0?0010?1?

Turgidodon_petiminis ???????????????1112101201001110000000?00112210110111000111010220100121011?0?0010?11

Turgidodon_praesagus ???????????????1112101201001(0 1)10000000100112210110111010111010220100121011?0?0010?11

Turgidodon_rhaister ???????000000???112101200001000000000000112210110111010111010220100121011?0?0010?11

Turgidodon_russelli ???????000000??01121012010011100000000002122101101110(0 1 2)01110102201001210111000010011

Varalphadon_crebreforme ????????????????1?23?110------000010????120210?????????????1012010012001110?0010?1?

Varalphadon_creber ????????????????112(1 3)01100-000000101011101202101101110001100????????1?????????????0?

Varalphadon_wahweapensis ???????????????0112(1 3)01100-00000010101110120210110111000110010220000121011?0?0010?0?

Unnuakomys_hutchisoni ???1???????????01122----(1 2)2-0(0 1)00011010100210010000111110112110220101121011100(0 1)011101

Heleocola_piceanus ???????????????????????????????????????????????????????????21220100121011?(0 1)?0011?2?

;

END;
