# Supplementary figures and images for: A new Late Cretaceous metatherian from the Williams Fork Formation, Colorado
Source: PLoS One. 2024 Oct 23;19(10):e0310948. doi: 10.1371/journal.pone.0310948 (PMC11498682; doi:10.1371/journal.pone.0310948)

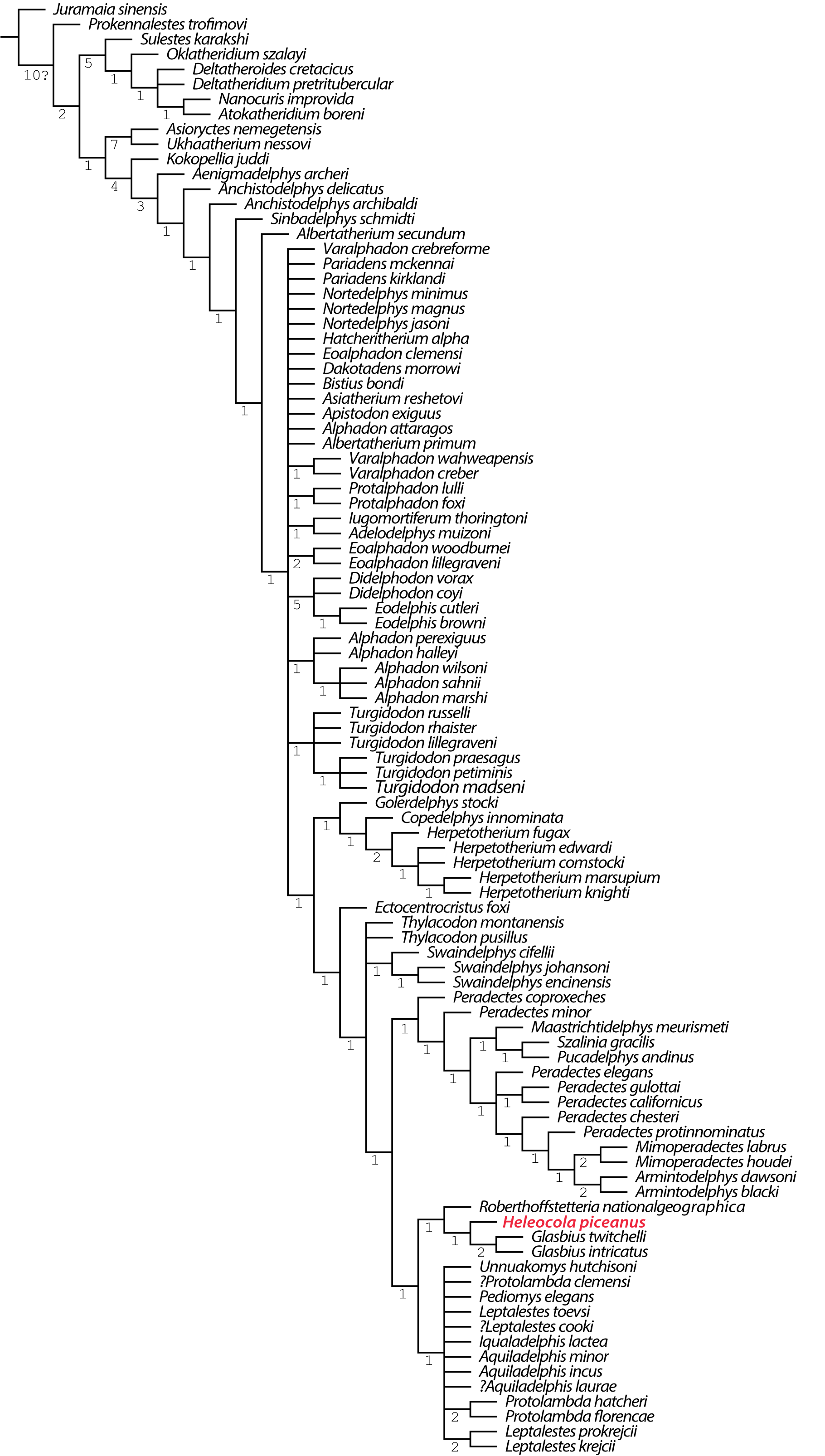

Supplement: S2 Fig — Heleocola piceanus (in red) is recovered as the sister taxon to Glasbius. Numbers at nodes correspond to Bremer branch supports calculated from a pool of 30,000 suboptimal trees of up to 10 steps longer than the shortest trees. (TIF) [file pone.0310948.s004.tif]
